# Expression of Irisin/FNDC5 in Cancer Cells and Stromal Fibroblasts of Non-Small Cell Lung Cancer

**DOI:** 10.3390/cancers11101538

**Published:** 2019-10-11

**Authors:** Katarzyna Nowinska, Karolina Jablonska, Konrad Pawelczyk, Aleksandra Piotrowska, Aleksandra Partynska, Agnieszka Gomulkiewicz, Urszula Ciesielska, Ewa Katnik, Jedrzej Grzegrzolka, Natalia Glatzel-Plucinska, Katarzyna Ratajczak-Wielgomas, Marzenna Podhorska-Okolow, Piotr Dziegiel

**Affiliations:** 1 Division of Histology and Embryology, Department of Human Morphology and Embryology, Wroclaw Medical University, 50-368 Wroclaw, Polandpiotr.dziegiel@umed.wroc.pl (P.D.); 2 Department of Thoracic Surgery, Wroclaw Medical University, 53-439 Wroclaw, Poland; 3 Department of Thoracic Surgery, Lower Silesian Centre of Lung Diseases, 53-439 Wroclaw, Poland; 4 Division of Ultrastructure Research, Wroclaw Medical University, 50-368 Wroclaw, Poland; 5 Department of Physiotherapy, Wroclaw University School of Physical Education, 51-612 Wroclaw, Poland

**Keywords:** irisin, Ki-67, TTF-1, p63, non-small cell lung cancer, cancer-associated fibroblasts, cancer

## Abstract

*Background:* Recent in vitro studies have indicated that irisin inhibits proliferation, migration and epithelial-mesenchymal transition. Irisin expression has not been studied in tumour tissues of non-small cell lung cancer (NSCLC) patients yet. The aim of the study was to determine the irisin expression in NSCLCs in comparison to the clinicopathological factors and expression of TTF-1, p63 and Ki-67. *Material and methods*: Tissue microarrays with 729 NSCLC and 140 non-malignant lung tissue (NMLT) were used to perform immunohistochemical reactions. Laser Capture Microdissection (LCM) was used to collect cancer and stromal cells from NSCLCs. *FNDC5* expression was tested for LCM samples, 75 NSCLCs and 25 NMLTs with the RT-PCR technique. Western-blot, immunofluorescence reaction and RT-PCR assays were performed on lung cancer cell lines. *Results*: Irisin expression was observed in NSCLC cancer cells and stromal fibroblasts. In cancer cells, irisin expression was decreased in higher grades (G) of malignancy, tumour size (T) and according to lymph node metastasis. In stromal cells, irisin expression was increased in higher G and advanced T. A shorter overall survival was observed in patients with higher irisin expression in NSCLC stromal cells. *Conclusions*: Irisin expression in stromal fibroblasts may influence cancer cell proliferation and may be a prognostic factor for survival in NSCLC.

## 1. Introduction

Non-small cell lung cancer (NSCLC), which accounts for 80% of all lung cancers, is still one of the tumours with the worst prognosis. Adenocarcinoma (AC) and squamous cell carcinoma (SCC) are the most important types of NSCLC [1]. Despite the development of new targeted therapies, NSCLC is still one of the leading causes of cancer-related deaths [2]. Due to poor prognosis among patients with NSCLC, there is still a need to search for more effective diagnostic and prognostic markers. The most recent studies have revealed that irisin could be such a marker, the expression of which was observed in NSCLCs in our study.

The irisin protein was detected for the first time in mouse skeletal muscle fibres by Boström et al. in 2012 [3]. In their research, they suggested that irisin is formed as the result of the post-translational modification of fibronectin type III domain-containing protein 5 (FNDC5). The protein results from the cleavage and glycosylation of the 112 amino acids of the FNDC5 chain. An elevated expression of irisin (myokine) was observed in the sarcolemma and cytosol of skeletal muscle fibres in response to physical activity [3,4]. It seems that irisin may be a link between physical exercise and changes in the metabolism of the adipose tissue [5,6]. It has been confirmed that this protein is expressed under the influence of the PGC-1*α* factor and is responsible for the conversion of white adipose tissue into brown tissue by increasing the expression of uncoupling protein 1 (UCP1) in the mitochondria [7], which is related to the adaptation of the body to low temperatures and the maintenance of a constant body temperature in hibernating animals. The uncoupling of oxidative phosphorylation is a means of generating heat to maintain body temperature instead of producing adenosine triphosphate (ATP) [8].

Subsequent studies on irisin revealed that it is also expressed in other normal tissues and organs, e.g. in the myocardium, the kidneys and the walls of blood vessels. The protein has also been detected in cancer cells, including cancer of the digestive system, breast and ovarian carcinomas [8,9,10,11,12]. However, it is unclear whether irisin affects endocrine cells (when released into the plasma) or paracrine cells (if it is secreted locally by tumour cells) [13].

It is believed that a local elevation of irisin expression in altered, cancerous tissues results in local hyperthermia. An increase in the local temperature can lead to the coagulation of proteins and the disruption of cell division by inhibiting the synthesis of ATP in the mitochondria. In addition, it can also destroy the blood vessels that nourish the tissues [14]. Lower levels of serum irisin were observed in patients with breast cancer when compared to the control group [15]. On the other hand, irisin added to the breast cancer cell lines resulted in an intensified cytotoxic effect of chemotherapeutics [16]. However, Shao et al. [17] observed in an *in vitro* study in lung cancer cells that irisin inhibits the proliferation, migration and epithelial-mesenchymal transition via the PI3K/AKT/Snail pathway. They also revealed that the protein is associated with a decreased Snail protein expression, which is responsible for the epithelial-mesenchymal transition (EMT) [17].

The level of irisin expression has not been studied in tumour tissues of NSCLC patients yet. The aim of this study was to detect the localization and the level of irisin expression, as well as the *FNDC5* gene, in NSCLCs and lung cancer cell lines. In addition, irisin expression was compared with clinicopathological factors to examine the significance of the protein as a prognostic and predictive marker in NSCLCs.

## 2. Results

### 2.1. Immunohistochemical (IHC) Detection of Irisin Expression in Tissue Microarrays (TMA) with NSCLC

**Figure 1:**
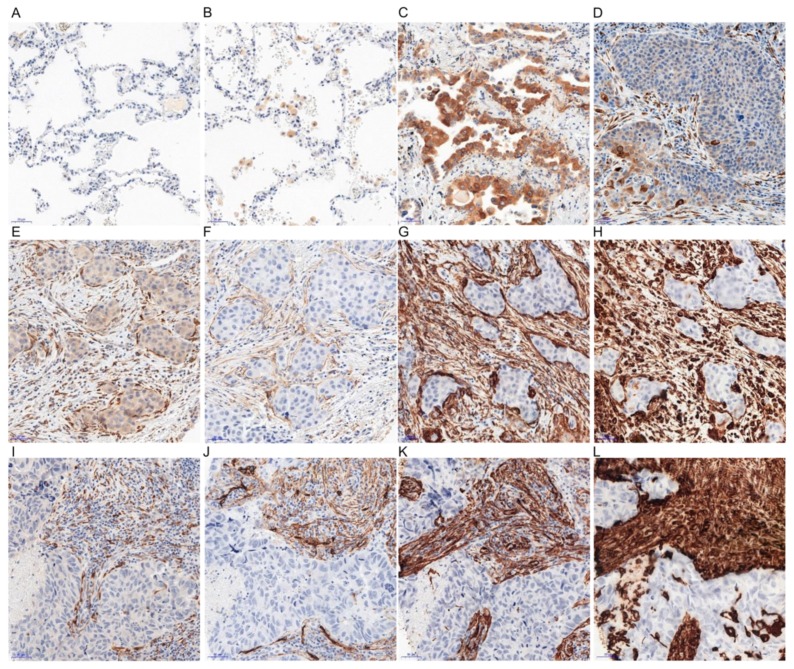
Positive immunohistochemical reactions (IHC - brown colour) indicating irisin expression performed on healthy lung tissue (**A**,**B**) as well as in different subtypes of NSCLC in AC cancer cells (**C**) and stromal cells (**E**), in SCC cancer cells (**D**) and in stromal cells (**I**). Lack of irisin expression—healthy lung tissue (**A**), irisin expression in macrophages (**B**). Comparison of irisin expression in cancer stroma with PDPN (in AC—**F**, in SCC—**J**), *α*SMA (in AC—**G**, in SCC—**K**) and Vimentin (in AC—**H**, in SCC—**L**) magnification, 200×.

**Figure 2:**
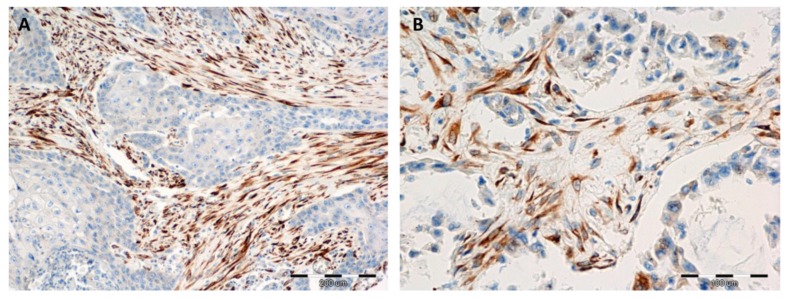
Positive immunohistochemical reactions (IHC—brown colour) indicating irisin expression in NSCLC stromal cells which are morphologically similar to fibroblasts, magnification ×200 (**A**), ×400 (**B**).

We did not find any expression of irisin in the epithelial cells of the normal lung parenchyma in 140 cases. We observed the expression of irisin in pulmonary macrophages (Figure 1). In contrast, in NSCLC tumours, the expression of irisin was observed in the cytoplasm of cancer cells and the cytoplasm of tumour stromal cells (Figure 2). Therefore, the expression of the protein was evaluated in both of the above-mentioned cell types (Table 1).

**Figure 3:**
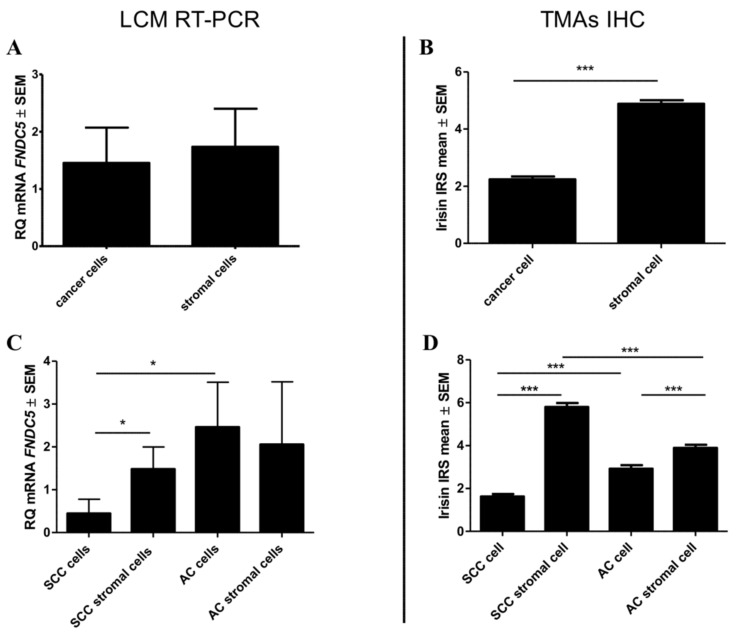
Comparison of mRNA FNDC5 expression levels collected by using Laser Capture Microdissection and detected by real-time PCR (**A**,**C**) with irisin expression levels detected by IHC reactions performed on Tissue Microarrays (**B**,**D**) in cancer cells and stromal cells of NSCLC (**A**, **B**) and according to subtypes: SCC and AC (**C**,**D**) *** *p* ≤ 0.001, * *p* ≤ 0.05.

The expression of irisin in both types of cells (cancer cells and stromal cells) was also compared in different subtypes of NSCLC: AC and SCC. The results obtained indicate a significant difference in the levels of irisin expression in cancer cells of SCC compared to the AC subtype (Mann-Whitney U, *p* < 0.0001) (Figure 3D).

A higher irisin expression was observed in the AC type (mean 2.9 ± 0.16) in comparison to the SCC one (mean 1.6 ± 0.12). The level of irisin expression in stromal cells was also different in both NSCLC subtypes (U-Mann-Whitney, *p* < 0.0001). A higher level was noticed in SCC (mean 5.8 ± 0.18) stromal cells in comparison to AC stromal cells (mean 3.8 ± 0.15).

### 2.2. mRNA FNDC5 Expression Level in NSCLC

**Figure 4:**
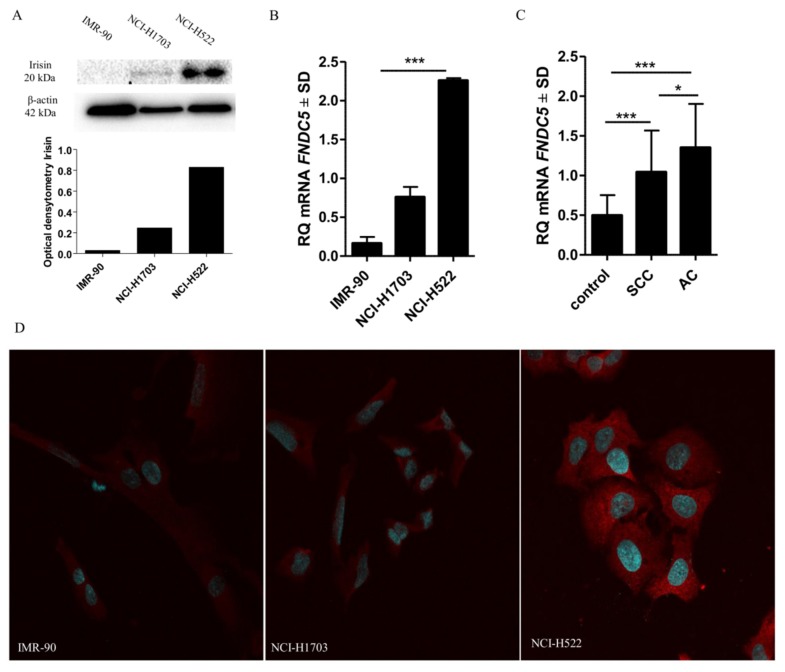
Comparison of irisin expression levels detected by Western-blot (**A**) and mRNA of FNDC5 (**B**) by confocal microscopy (**D**) in normal fibroblast (IMR-90) and different cancer cell lines (NCI-H1703, NCI-H522). Comparison of mRNA FNDC5 expression levels detected by real-time PCR in healthy lung tissues and different subtypes of NSCLC (**C**) ** *p* ≤ 0.005, *** *p* ≤ 0.001.

RT-PCR revealed a higher expression of FNDC5 mRNA in tissues of NSCLC tumours (mean 31.36 ± 5.6) than in NMLTs (mean 3.6 ± 0.3) (Mann-Whitney U, *p* < 0.0001). We also observed a higher mRNA expression in the major subtypes of NSCLC (SCC and AC) than in the normal lung tissue (Mann-Whitney U, *p* < 0.0001, in both cases). Moreover, we noticed that the expression of the gene was higher in AC tumours (mean 40.73 ± 9.5) than in SCC ones (mean 24.51 ± 6.9). The difference between AC and SCC was significant (Mann-Whitney U, *p* = 0.0208). A graphic comparison of the gene expression in tumours and in the control tissue is presented in Figure 4C.

The abovementioned observations were confirmed with the use of LCM. The expression of the gene was higher in AC cells (mean 2.5 ± 1.0) in comparison to SCC cells (0.4 ± 0.3) (Figure 3C). Additionally, the results achieved by using LCM indicated that FNDC5 mRNA expression was noticed both in lung cancer cells (mean 1.4 ± 0.5) and in stromal cells (mean 1.7 ± 0.6) of NSCLC. The expression level of the *FNDC5* gene was higher in NSCLC stromal cells in comparison to NSCLC cancer cells and normal lung cells (mean 1.2 ± 0.3). Moreover, we noticed a significantly decreased gene expression in SCC cells in comparison to SCC stromal cells (mean 1.5 ± 0.4) and normal cells (mean 1.3 ± 0.3; respectively, Mann-Whitney U *p* = 0.047; *p* = 0.026).

### 2.3. Comparison of the Expressions of Irisin with Vimentin, Alpha-smooth Muscle actin (αSMA) and Podoplanin (PDPN) Expression

We observed the expression of irisin in tumour stromal cells, which resemble fibroblast morphology (Figure 1). The IHC reactions were conducted on serial sections of tissues, detecting the expression of irisin, vimentin, *α*SMA and PDPN. We noticed a similar pattern of expression of the above markers in NSCLC tumour stroma. A comparison of the expressions of the markers is shown in Figure 1E–L.

### 2.4. Associations between Irisin and Cancer Cell Proliferation

**Figure 5:**
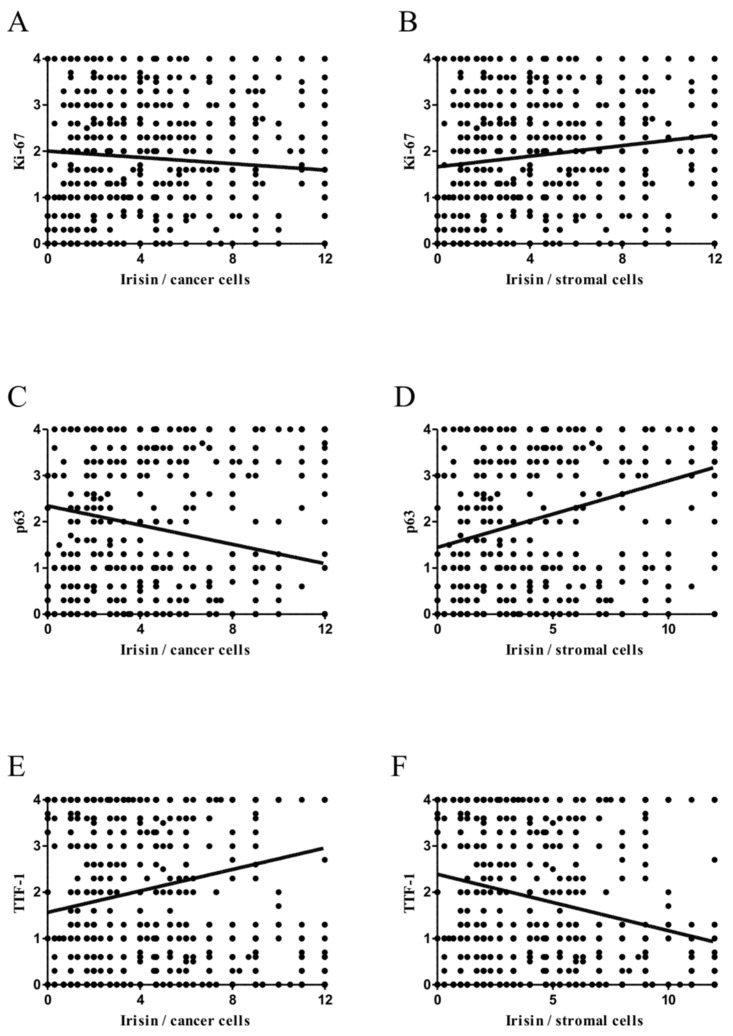
Correlations of irisin expression levels (cancer cells—**A**,**C**,**E**; stromal cells—**B**,**D**,**F**) with diagnostic markers Ki-67 (**A**,**B**), p63 (**C**,**D**) and TTF-1 (**E**,**F**) expression levels in NSCLC cells.

The analysis of the results suggests that the effect of irisin expression on NSCLC cell proliferation may be dualistic and depends on which cells (cancer or stromal cells) irisin expression is observed. Irisin expression in NSCLC cells correlated weakly negatively with the expression of the Ki-67 antigen (*r* = -0.11, *p* < 0.0001). The opposite trend was observed in the case of irisin expression in the cancer stroma (Figure 5A,B). It correlated weakly positively with the Ki-67 antigen expression level detected in the nuclei of lung cancer cells (r = 0.19, *p* < 0.0001).

### 2.5. Comparison of Irisin with TTF-1 and p63 Expression Levels

We observed nuclear expression of TTF-1 and p63 in NSCLC cells. We examined the relationship between the expression level of irisin and the above markers (Figure 5). We noticed a weak positive correlation of irisin expression level with TTF-1 in tumour cells (*r* = 0.21, *p* < 0.0001) and a weak negative correlation with p63 (*r* = −0.20, *p* < 0.0001). In contrast, the expression of irisin in the cancer stroma correlated weakly positively with p63 (*r* = 0.28, *p* < 0.0001) and weakly negatively with TTF-1 (*r* = −0.23, *p* < 0.0001).

### 2.6. Associations between Irisin Expression in Cancer Cells and Clinicopathological Parameters

**Figure 6:**
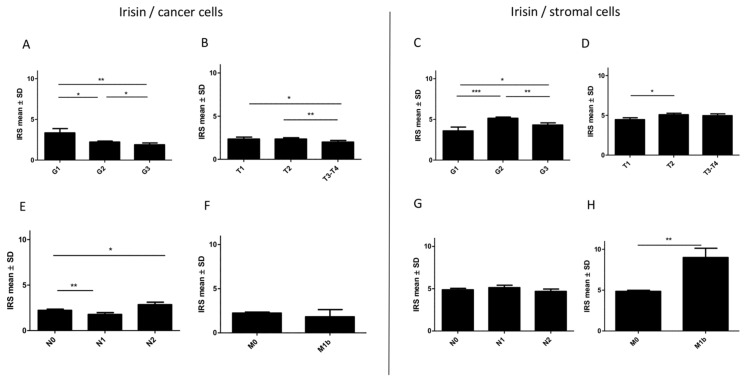
Comparison of irisin expression levels in NSCLC cells according to the grade of malignancy (**A**), tumour size (**B**), lymph node status (**E**) and metastases (**F**). Comparison of expression levels of irisin in stromal cells of NSCLC according to the grade of malignancy (**C**), tumour size (**D**), lymph node status (**G**) and metastases (**H**) * *p* ≤ 0.05, ** *p* ≤ 0.005, *** *p* ≤ 0.001.

The relationship of irisin expression levels in cancer cells with the clinicopathological parameters in NSCLC is presented in Table 1 and Table 2. In cancer cells, the level of expression of irisin decreased in higher grades (G) of malignancy (Kruskal-Wallis, *p* = 0.008). The mean level was 3.3 ± 0.52 in G1, 2.2 ± 0.11 in G2 and 1.8 ± 0.23 in G3. The differences between the groups were statistically significant (Figure 6A). The expression level of irisin also decreased in higher tumour sizes (T) (Kruskal-Wallis, *p* = 0.038). We observed the highest mean level of irisin in T1 (2.35 ± 0.21). The mean value was 2.34 ± 0.15 in T2 and 1.9 ± 0.18 in T3 (Figure 6B).

We also observed changes in irisin expression in relation to lymph node metastases (Kruskal-Wallis, *p* = 0.0091). The highest expression of irisin was detected in tumours with mediastinal lymph node metastases (N2) (Figure 6E). The level of irisin in the N2 group (mean 2.8 ± 0.28) was significantly higher than in the group without lymph node metastases (N0, mean 2.2 ± 0.12) and in the group with metastases to hilar and intrapulmonary lymph nodes (N1, 1.7 ± 0.20) (Mann-Whitney U, *p* = 0.0124; *p* = 0.011, respectively). Additionally, we noticed a lower mean expression of irisin in the group with distant metastases (M1b, mean 1.8 ± 0.81) in comparison to the non-metastatic group (M0, mean 2.2 ± 0.10). However, the difference between M0 and M1b was not statistically significant.

### 2.7. Associations between Irisin Expression in Stromal Cells and Clinicopathological Parameters

The relationship of the irisin expression level in tumour stromal cells with the clinicopathological parameters in NSCLC is presented in Table 1 and Table 2. The level of irisin expression in stromal fibroblasts was different between grades of malignancy (Kruskal-Wallis, *p* = 0.0003). The level of protein expression increased in G2 (mean 5.14 ± 0.14) compared to G1 (mean 3.59 ± 0.46). However, in G3 (mean 4.31 ± 0.26) it was lower than in G2 (Figure 6C). The level of irisin expression also increased in advanced pTs. The level was significantly higher in T2 (mean 5.07 ± 0.18) than in T1 (mean 4.45±0.23Mann-Whitney U, *p* = 0.0228). However, the difference between T2 and T3–4 (4.96 ± 0.22) was unimportant. Changes in irisin expression in relation to the pN status (Kruskal-Wallis, *p* = 0.5202) were statistically insignificant. A higher protein expression was observed in patients from the M1b group (mean 9.0 ± 1.1) compared to M0 (mean 4.85±0.12) (Mann-Whitney U, *p* = 0.0054).

### 2.8. Associations between Irisin Expression and the Overall Survival (OS)

**Figure 7:**
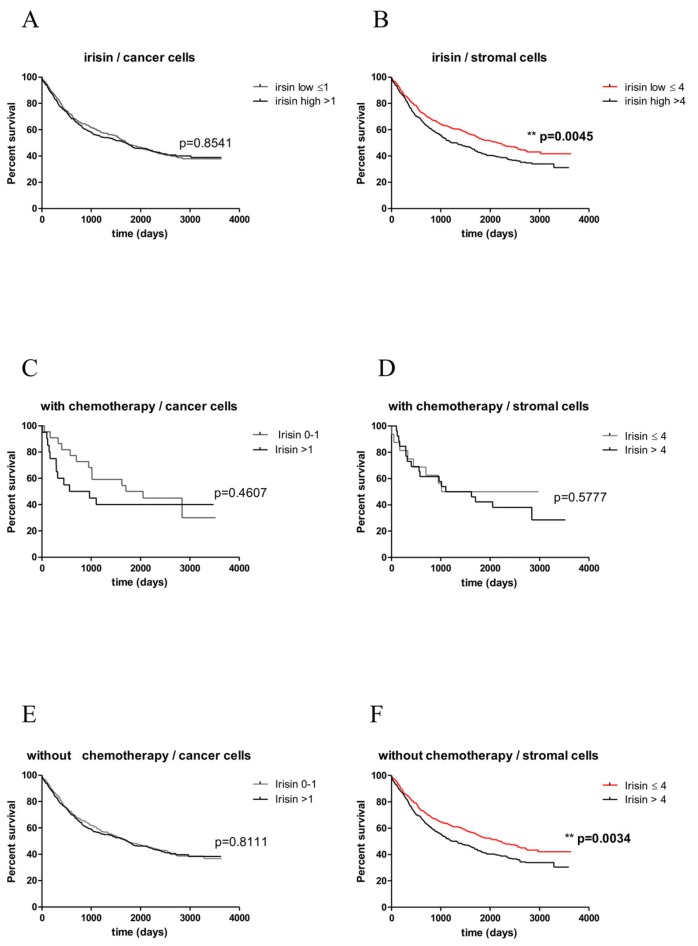
Kaplan-Meier survival curves presenting the prognostic impact of irisin expression levels detected by immunohistochemistry (IHC) in cancer (**A**,**C**,**E**) and stromal cells (**B**,**D**,**F**) on overall survival (OS) of patients with non-small cell lung cancer (NSCLC). Patients were grouped according to the median value of the immunohistochemical expression of irisin in cancer and stromal cells of NSCLCs. The prognostic impact of irisin expression levels was studied in the whole patient group (**A**,**B**) and according to whether they received (**C**,**E**) or did not receive (**D**,**F**) preoperative chemotherapy.

We did not observe any relationship between the expressions of irisin in cancer cells and the OS (Figure 7A). A shorter OS was observed in patients with higher irisin expression in NSCLC stromal cells (*p* = 0.0045) (Figure 7B). Patient survival curves are shown in Figure 7. Additionally, the prognostic impact of irisin expression levels was studied in patients group who received or not received preoperative chemotherapy (Figure 7C–F). A shorter OS was observed in patients with higher irisin expression in NSCLC stromal cells (*p* = 0.0034). The results of the univariate and multivariate analyses are presented in Table 3. The multivariate analysis revealed that a high irisin expression in stromal cells was related to a shorter OS. However, the expression of irisin in cancer cells was not related to OS. Additionally, age ≥60 years, male sex, advanced pT, pN status and the stage of NSCLC were related to a shorter OS.

### 2.9. Comparison of Irisin Expression Levels in Cancer Cell Lines

Our *in vitro* study revealed a higher level of FNDC5 mRNA in cells of lung cancer lines NCI-1703 and NCI-H522 compared to the level in lung fibroblast cell line IMR-90. However, a statistically significant difference was observed only between the NCI-H522 lung AC cells and the IMR-90 cells (unpaired *t*-test, *p* = 0.0001—Figure 3A). The levels of irisin were also higher in the cells of both lung cancer lines as compared to the controls (Figure 3B). The results obtained were confirmed by confocal microscopy, western blot and RT-PCR (Figure 3A–B,D).

## 3. Discussion

Our study is the first in which the expression of irisin was evaluated in tumour tissues of NSCLC patients. So far, irisin expression has been investigated only on cell lines of lung cancer [17]. In addition, we conducted the study on a large group of 729 cases of NSCLC. Our study has revealed that irisin expression does not occur in normal lung epithelia. However, we have detected the presence of this protein not only in lung cancer cells but also in stromal cells.

The majority of previous studies were conducted using cell lines [17,18] instead of material obtained from patients. However, previous *in vitro* studies also confirmed the expression of irisin in lung cancer cells [17]. The IHC method on clinical tissue material was studied only by Kulogulu et al. [13] and Celik et al. [10] who observed an increased expression of irisin in breast, ovarian and cervical cancer cells and Aydin et al. [8] in gastrointestinal tract cancers. In our study, we revealed that both the level of expression of irisin in cancer cells and the expression of irisin in stromal cells were related to clinicopathological parameters such as pT, pN status and the grade of malignancy. Previous studies did not compare the expression of irisin with clinicopathological parameters. This is the first study describing this relationship.

Irisin expression in tumour stromal cells has not been fully described yet. In the light of the results we have obtained, the expression in stromal cells may have more prognostic significance than the expression observed in NSCLC cells. We noticed that a high irisin expression in fibroblasts is associated with worse patient prognosis and may be an independent prognostic factor. Perhaps the expression of irisin in the cancer stroma is typical only of lung cancer. Irisin is also expressed in healthy individuals and patients. Although muscle cells are the main cells that produce irisin, other normal cells can express irisin with variable degrees of intensity [13]. Cancer cells co-exist with many types of stromal cells, participating in the development of the cancer microenvironment. The main components of stromal cells include inflammatory cells (lymphocytes, granulocytes and macrophages), endothelial cells of blood and lymph vessels, pericytes and fibroblasts. One of the important components present in the tumour stroma are also fibroblasts recruited into cancer tissue, which are known as cancer-associated fibroblasts (CAFs).

These fibroblasts are thought to promote cancer progression by secreting various growth factors, chemokines and cytokines. These factors affect the proliferation, migration and invasiveness of tumour cells [19]. PDPN and αSMA are the proteins whose expression is elevated in CAFs. Due to the fact that irisin expression was observed in cells that resembled fibroblasts morphologically, we compared it with the expression of PDPN as well as with vimentin and αSMA. We detected a very similar pattern of irisin expression in stromal cells, PDPN, αSMA and vimentin. This examination may suggest that stromal cells expressing irisin are CAFs. PDPN which is present in CAF stroma, is associated with a worse patient prognosis [20], which is in line with our study.

It is also believed that stromal fibroblasts can affect tumour growth by means of a variety of mechanisms such as angiogenesis, recruitment of endothelial progenitor cells and changes in the extracellular matrix. According to one of the hypotheses, stromal fibroblasts can affect cancer cell migration [21]. Several studies suggest that irisin may participate in the regulation of epithelial-to-mesenchymal transition (EMT) in different type of cancers. Studies conducted by Shao et al. [17] indicated that irisin triggered the inhibition of migration and invasion of the lung cancer A549 and NCI-H446 cell lines. Liu et al. [18] used the MTT method, scratch wound healing and transwell assays in order to observe that in the case of the pancreatic cancer cell line, irisin also inhibited cell growth, migration and EMT. The authors suggested that the inhibition of EMT with irisin was via the activation of the adenosine monophosphate-activated protein kinase (AMPK), which affects the downregulation of the mTOR pathway [18]. On the other hand, Shi et al. [22] described a positive effect on the proliferation and invasion of liver cancer cells targeting the PI3K/AKT pathway. However, Moon et al. [23] did not observe any effect of irisin on the proliferation or migration of oesophageal, thyroid, colon or endometrial cancer cells. These contradictory results of in vitro studies with irisin may be due to the use of a glycosylated or non-glycosylated form of the protein, which can cause different biological activities of irisin [18]. The irisin we have observed is produced by cells and is therefore a glycosylated form. In our study, the influence of irisin on proliferation revealed that its expression in the stroma correlates positively with the expression of the Ki-67 antigen in cancer cells. In addition, we observed that the expression of irisin in stromal cells was higher in larger tumours (T) and a higher grade of malignancy (G). On the contrary, the expression of irisin in cancer cells was lower with a higher grade of malignancy and pT status. These observations suggest that irisin in stromal cells may influence the development and proliferation of lung cancer. In contrast, irisin expressed by cancer cells may inhibit cancer progression.

Apart from its involvement in proliferation and migration, irisin is primarily associated with metabolic changes in the adipocytes [17]. The most characteristic metabolic feature of cancer cells is their ability to consume glucose rapidly through aerobic glycolysis resulting from the rapid proliferation of these cells [24]. Glucose production occurs as a result of glycolysis regardless of the availability of oxygen. Alterations in cancer cell metabolism are essential to generate and maintain carcinogenesis. Recent studies have indicated a dynamic correlation between glycolysis, mitochondrial function and synthesis pathways in cancer cells [25,26]. Irisin is expressed in normal body tissues in cells which have the highest energy demand, such as skeletal miofibers, heart muscle cells or hepatocytes [3,4]. In addition, irisin is associated with glucose homeostasis and improves glucose uptake by activating AMPK*α*2-mediated p38 MAPK in muscle cells [27]. This may explain a higher expression of irisin in lung cancer cells that also have a high demand for glucose uptake due to tumour growth. Moreover, Lee et al. [27] observed that muscle cells relocate the glucose transporter GLUT-4 into the cell membrane under the influence of irisin. On the other hand, irisin is responsible for increasing the expression of UCP1, which inhibits ATP synthesis in the mitochondria and increases the local temperature [7,14]. The decreased level of ATP is associated with the activation of AMPK and the downregulation of the mTOR pathways. The AMPK-mTOR pathway plays a pivotal role in the growth of cancer cells. These processes may affect the proliferation of cancer cells and could explain why a strong expression of irisin in cancer cells is related to tumor repression [18,28]. It could be assumed that in the first step of the disease, cancer cells have an increased expression of irisin and this could be associated with changes in metabolism and as well as the biogenesis of mitochondria, because this protein is involved in such process. However, at later stages of the disease irisin could be down-regulated because of its influence on UCP1 expression and reduction of ATP synthesis. In our study, we also observed that irisin expression decreased with the progression of the disease in cancer cells while, in contrast, the expression increased in fibroblasts.

The surrounding stromal cells could also participate in the process of reprogramming tumour cell metabolism. The role of CAFs in the metabolism of cancer cells was described as the Reverse Warburg effect. According to this hypothesis, tumour cells together with the adjacent microenvironment activate tumour stromal fibroblasts in the presence of certain factors. In this way, cancer cells control stromal fibroblasts and take advantage of their metabolism [24,29]. This could explain an increased expression of irisin in lung cancer stromal cells and elucidate why a higher expression in these cells results in an increased proliferation and cancer progression as reflected in our study. Fiaschi et al. [30] observed that CAFs undergo Warburg reprogramming and mitochondrial oxidative stress. These processes occurred in fibroblasts under the influence of prostate cancer cells.Intracellular activation was observed by up-regulation of the glucose GLUT1 transporter, just to name an example. In addition, irisin expression is controlled by the PGC-1*α* factor [7], which is associated with mitochondrial biogenesis and can be activated by mitochondrial oxidative stress [31]. Therefore, the following question arises: can irisin in NSCLC stromal cells be involved in reprogramming the metabolism of these cells under the influence of cancer cells? In our study, its high level in stromal cells was certainly associated with tumour development and a worse patient survival. However, further studies are needed to clarify this.

Additionally, it has not been fully explained whether the elevation of irisin expression in cancer cells is associated with its local or systemic production and the mechanism of its secretion into the bloodstream [13]. There are some contradictory studies on the serum level of irisin detected by using ELISA tests. The levels in renal cancer and polycystic ovary syndrome patients were significantly higher compared to the control group [32,33]. In contrast, the serum irisin level in breast cancer patients was lower than in the control group [15]. Liu J et al [18] noticed irisin binding to cell surface receptors in *in vitro* studies.

However, we observed a cytoplasmic IHC reaction that detected irisin in tissues with NSCLC. We also examined the mRNA FNDC5 expression level that encodes the protein from which irisin is cleaved. The expression of the *FNDC5* gene was significantly higher in cancer cells compared to normal lung tissue. This suggests that cancer cells produce irisin, but it cannot be excluded that cancer cells could also incept irisin from the microenvironment. Laser Capture Microdissection and RT-PCR, which were used to determine the FNDC5 mRNA expression levels in NSCLC stromal and cancer cells, confirmed the TMA IHC observations. We noticed a higher *FNDC5* gene expression in NSCLC stromal cells in comparison to cancer cells. Moreover, Giaginis et al. [12] conducted a study on the mRNA expression of the *FNDC5* gene in normal hepatocytes and obtained from patients with liver cancer. The expression of *FNDC5* mRNA was higher in liver cancer cells. However, Giaginis et al. also detected a strong transcriptional relationship with the genes related to lipogenesis, inflammation and tumourigenesis, such as *SREBF-1, SCD-1, TNF, IL-6* and *NOTCH1*. This relationship indicates the potential participation of irisin in the processes of lipogenesis, inflammation and tumourigenesis [12].

Summing up our findings, an elevated irisin expression level in cancer cells and tumour stromal fibroblasts of NSCLC patients was observed for the first time. Our study indicates that the expression of irisin in stromal fibroblasts may be associated with an increased proliferation of cancer cells and may also be an independent prognostic factor for survival in patients with NSCLC. The level of expression in cancer cells was higher in AC cells compared to SCC. However, irisin expression in stromal cells was higher in SCC.

## 4. Materials and Methods

### 4.1. Patient Cohort

From 2007 to 2011, patients with NSCLC underwent major resection of lung parenchyma or sublobar resection at the Department of Thoracic Surgery of Wroclaw Medical University, Poland. The study material consisted of 729 (including ACs *n* = 348 and SCCs *n* = 381) paraffin blocks and 75 frozen samples of NSCLC, as well as 140 embedded in paraffin and 25 frozen samples of non-malignant lung tissue (NMLT). The histopathological evaluation was made according to the World Health Organization criteria and the pathological staging was standardized according to the 8th TNM edition [34]. Patient characteristics are presented in Table 1. An informed written consent was obtained from all patients. The study was approved by the Bioethics Committee of the Wroclaw Medical University (ID No. KB-521/2017).

### 4.2. Cell Line Culture

Lung cancer cell lines NCI-H1703 (SCC) and NCI-H522 (AC), were obtained from the American Type Culture Collection (ATCC, Manassas, VA, USA) and used to perform molecular studies. The lung fibroblast cell line IMR-90 was used as the control. Lung cancer cells were cultured in RPMI-1640 medium (Lonza, Basel, Switzerland) and IMR-90 fibroblasts were cultured in EMEM medium (Lonza). The media were supplemented with 1% penicillin/streptomycin, L-glutamine and 10% foetal bovine serum (FBS). Cultures were carried out in the HERA cell incubator (Heraeus, Hanau, Germany) under constant conditions of 37 °C, 5% CO_2_ concentration and a 95% level of humidity.

### 4.3. Tissue Microarray (TMA) Immunohistochemical (IHC) Reactions

Tissue microarrays (TMAs) were prepared from 729 NSCLC and 140 NMLT sections. Haematoxylin and eosin stained slides were prepared and scanned with the use of the Pannoramic Midi II histological scanner (3D HISTECH Ltd, Budapest, Hungary). Three representative cancer sites were selected by the Pannoramic Viewer (3D HISTECH Ltd., RRID: SCR_014424) Software. Subsequently, the selected cancer sites were transferred with a core of 1.5 mm to the tissue arrays using the TMA Grand Master (3D HISTECH Ltd.).Immunohistochemical reactions were performed on sections of each TMA.

After deparaffinization and hydration, thermal epitope demasking was done using low pH Target Retrieval Solution (Agilent Technologies, Santa Clara, CA, USA) for 20 min at 97 °C in a Dako PT Link (Dako, Glostrup, Denmark) apparatus. In order to detect protein expression, specific primary antibodies were used: polyclonal rabbit anti-irisin/FNDC5 (dilution 1:50; code no. NBP2-14024; Novus Biologicals, Littleton, CO, USA), monoclonal mouse anti-Ki-67 antibody (ready to use, Clone MIB-1, code IS626; Dako), anti-TTF-1 (ready to use, Clone 8G7G3/1, code IR056; Dako), anti-p63 (ready-to-use, Clone DAK-p63, code IR662; Dako), anti-Podoplanin (ready to use, clone D2-40 (PDPN), code ISO072; Dako), anti-Vimentin (ready to use, clone V9, code GA630; Dako) and anti-αSMA (ready to use, clone IS611, code 1A4; Dako). An automatic system DAKO Autostainer Link48 (Dako) and an EnVision FLEX kit (Dako) were used to visualize the antigens.

The evaluation of the IHC reactions was conducted by two independent pathologists-researchers (Piotr Dziegiel and Katarzyna Nowinska). The assessment was done at ×200 magnification with the use of a BX41 (Olympus, Tokyo, Japan, RRID:SCR_017022) light microscope coupled with visual circuit and the CellD (Olympus) software. The expression levels of nuclear proteins TTF-1, p63 and Ki-67 were determined with the use of the five-point evaluation scale (0-no expression, 1 point—1%–10%, 2 points—11%–25%, 3 points—26%–50%, 4 points >50%) [35]. For the evaluation of the cytoplasmic expression of irisin level, the semiquantitative method immunoreactive score (IRS) according to Remmele and Stegner was used [36]. The final result was the product of the scores obtained by the estimation of the intensity (1—weak, 2—moderate, 3—strong) of the colour reaction and the percentage of IRS-positive cancer cells (0-no expression, 1 point—1%–10%, 2 points—11%–50%, 3 points—51%–80%, 4 points >80%).

### 4.4. Laser Capture Microdissection (LCM)

Frozen tissue samples from 10 NSCLC (5 SCC, 5 AC) cases and 6 NMLT ones were used for RNA extraction. In order to compare the expression level of irisin in both cancer and stromal cells from the 10 NSCLCs, they were collected separately. Tissue sections of 10 µm of thickness were made using a Leica CM1950 cryostat (Leica Microsystems, Wetzlar, Germany) and placed on a polyethylene terephthalate membrane slide (MMI, Glattbrugg, Switzerland). The MMI CellCut Plus System (MMI) was used to perform LCM. The dissected samples of cancer and stromal cells of NSCLCs and NMLTs were collected onto an adhesive lid of 500 µL tubes (MMI). Total RNA was isolated using the RNeasy Micro Kit (Qiagen, Hilden, Germany) and cDNA was synthesized using the QuantiTect Reverse Transcription Kit (Qiagen), and finally Real-time PCR reactions were performed.

### 4.5. Real-Time PCR (RT-PCR)

The LCM samples from cancer cells and stromal cells of NSCLC and NMLT were used for RT-PCR. Additionally, RT-PCR reactions were performed for 75 sections of NSCLC, 25 control sections of NMLT, lung cancer cell lines NCI-H1703 and NCI-H522 and the normal fibroblast cell line IMR-90. RNA isolation was done using the RNeasy Mini Kit (Qiagen), and The High-Capacity cDNA Reverse Transcription Kit with RNase Inhibitor (Applied Biosystems, Waltham, MA, USA) was used to perform the reverse transcription reaction. The expression level of *FNDC5* (*FNDC5*; TaqMan Gene Expression Assay, Applied Biosystems) was tested using the 7900HT Fast Real-Time PCR System (Applied Biosystems) and the relative quantification (RQ) method. The analysis of FNDC5 gene expression was done using the RQ Manager 1.2 software (Applied Biosystems). The results were standardized in relation to the reference gene of β-actin expression (*ACTB*; TaqMan Gene Expression Assay, Applied Biosystems). Changes in the level of *FNDC5* gene expression in NSCLC cells were assessed in relation to normal lung cells. The evaluation of *FNDC5* gene expression by real-time PCR was repeated three times.

### 4.6. Western-Blot Analysis

For each analysis, 4 × 10^6^ NCI-H1703, NCI-H522 and IMR-90 cells in the exponential growth phase were taken. The extraction of whole cell proteins was made using the RIPA buffer [50 mM Tris HCl; 150 mM NaCl; 0.1% SDS; 1% Igepal (CA-630, Merck, Darmstadt, Germany); 0.5% sodium deoxycholate; protease inhibitor cocktail (Merck); 0.5 mM PMSF]. The concentration of proteins was determined using the Pierce BCA Protein Assay Kit (Thermo Fisher Scientific, Waltham, MA, USA). The proteins were denatured in sample loading buffer (250 mM Tris-HCl, 40% glycerol, 20% *β*-mercaptoethanol, 8% SDS and bromophenol blue), transferred to a PVDF membrane (Millipore, Burlington, MA, USA) and blocked with 2% non-fat milk (Bio-Rad, Marnes-la-Coquette, France) in TBST 0.1% for 1 hour at room temperature. Subsequently, the membrane was incubated with rabbit polyclonal anti-irisin/FNDC5 antibody diluted in 0.5% milk in TBST 0.1% (1:200, code no. NBP2-14024, Novus Biologicals, Centennial, CO, USA) overnight at 4 °C with a gentle shaking. Next, incubation with secondary horseradish peroxidase conjugated with donkey anti-rabbit antibody diluted in milk 0.5% in TBST 0.1% (1:3000, code no. 711-035-052; Jackson ImmunoResearch, Cambridgeshire, UK) was performed for 1 hour at room temperature. The proteins were visualized using the Luminata Classico Western HRP Substrate (Millipore). The membrane was stripped and incubated again, with monoclonal mouse anti-actin antibody (1:500, clone AC-40, code no. A4700, Merck) used as the control of protein amount loading. The data were collected in the Chemi-Doc XRS Molecular Imager apparatus (Bio-Rad). The optical density of the protein band was measured with the use of the Image Lab (Bio-Rad) software. The experiment was repeated three times.

### 4.7. Confocal Microscopy

In order to prepare 24-h microcultures cells were set up on slides with Millicell EZ 8-well glass slides (Merck). For microculture, 600 μL of 33 × 10^4^ cells/mL suspension was instilled into wells on the slides and placed in an incubator at 37 °C for 24 h. After the incubation, the cells were fixed with the use of 4% formaldehyde and the immunofluorescence (IF) reactions were performed with the use of the specific polyclonal rabbit anty-irisin/FNDC5 antibody (dilution 1:50; code no. NBP2-14024; Novus Biologicals) at 4 °C overnight. Next, the preparations were incubated for 1 h with donkey anti-rabbit secondary AlexaFluor 568 conjugated antibody (dilution 1:2000; clone, code no.; Invitrogen, Carlsbad, CA, USA) in the reagent with background-reducing component (Agilent Technologies) and were mounted using the Prolong DAPI Mounting Medium (Invitrogen). The observations were made at x600 magnification with the use of a Fluoview FV3000 confocal microscopy (Olympus) coupled with the CellSense software (Olympus, RRID:SCR_016238).

### 4.8. Statistical Analysis

The Kruskal-Wallis test was used to evaluate the association between the intensity of irisin expression and the NSCLC subtypes. The Mann-Whitney U and Chi^2^ tests were used to estimate the clinicopathological factors of the NSCLC samples. Overall survival (OS) was measured from the date of surgery to the date of death or the last follow-up. The Kaplan-Meier analysis and the log-rank test were used to verify the relationship between the intensity of irisin expression and the patient survival. The Cox proportional hazard regression model was used to evaluate the clinicopathological characteristics related to OS (hazard ratio—HR and 95% confidence intervals—CIs). The statistical significance of the differences in the mRNA expression of the FNDC5 gene in the control tissues and the NSCLC ones was determined using the Mann-Whitney U test. The unpaired t-test was used to assess the differences between the level of irisin and FNDC5 mRNA expression in each cell line. The Spearman’s rank correlation was used to evaluate the association between the expression of the Ki-67 antigen, TTF-1 and p63 and that of irisin in cancer cells and stroma. The results were considered statistically significant if two-sided p values were at ≤0.05. The statistical analysis was made using Prism 5.0 (GraphPad, La Jolla, CA, USA, RRID:SCR_002798) and Statistica 13.1 (StatSoft, Cracow, Poland, RRID:SCR_014213).

## 5. Conclusions

In our study, we observed for the first time an elevated level of irisin expression in cancer cells and tumour stromal fibroblasts in tumour tissues of NSCLC patients. The level of expression in cancer cells varies between NSCLC subtypes and is higher in AC cells compared to SCC. In contrast, irisin expression in stromal cells is higher in SCC. Our study indicates that the expression of irisin in stromal fibroblasts may promote an increased proliferation of cancer cells and may as well be an independent prognostic factor for survival in patients with NSCLC.

## Figures and Tables

**Table 1:** Clinicopathological characteristics of NSCLC patients related to irisin expression in cancer cells and stoma cells.

Clinicopathological Parameter	n 729 (%)	Irisin Expression in NSCLC Cancer Cells			Irisin Expression in NSCLC Stromal Cells		
		Low 1–4	High 5–12	*Chi^2^Test* *p* Value	Low 1–4	High 5–12	*Chi^2^Test* *p* Value
Age ≤60	290 (39.8)	248 (85.5)	42 (14.5)	0.3911	155 (53.4)	135 (46.6)	0.7797
> 60	439 (60.2)	365 (83.1)	74 (16.9)		230 (52.4)	209 (47.6)	
Sex Male	540 (74)	458 (84.8)	82 (15.2)	0.3644	275 (50.8)	265 (49.2)	0.0846
Female	189 (26)	155 (82)	34 (18)		110 (58.2)	79 (41.8)	
Smoking Status Yes	631 (86.5)	535 (84.8)	96 (15.2)	0.1909	325 (51.5)	306 (48.5)	**0.0475**
No	98 (13.5)	78 (79.6)	20 (20.4)		61 (62.2)	37 (37.8)	
Histology Type AC	348 (47.6)	273 (78.5)	75 (21.5)	**<0.0001**	221 (63.5)	127 (36.5)	**<0.0001**
SCC	381 (52.4)	340 (89.2)	41 (10.8)		164 (43)	217 (57)	
Tumour Size (T) T1	177 (24.3)	148 (83.5)	29 (16.4)	**<0.0001**	102 (57.5)	75 (42.5)	0.3027
T2	329 (45.1)	272 (82.8)	57 (17.2)		166 (50.4)	163 (49.6)	
T3–4	223 (30.6)	120 (53.7)	103 (46.3)		117 (52.5)	106 (47.5)	
Lymph Nodes (N) N0	474 (65)	400 (84.4)	74 (15.6)	**0.0076**	252 (53.2)	222 (46.8)	0.3235
N1	134 (18.4)	121 (90.3)	13 (9.7)		64 (47.8)	70 (52.2)	
N2	121 (16.6)	92 (76)	29 (24)		69 (57.2)	52 (42.8)	
Metastasis (M) M0	723 (99.2)	608 (84.1)	115 (15.9)	0.9595	723	0	**<0.0001**
M1b	6 (0.8)	5 (83.3)	1 (16.7)		0	6	
Stage I	267 (36.6)	113 (42.3)	154 (57.7)	**<0.0001**	150 (56.2)	117 (43.8)	0.1049
II	245 (33.6)	213 (86.9)	32 (13.1)		116 (47.3)	129 (52.7)	
III–IV	217 (29.8)	176 (81.1)	41 (18.9)		119 (54.8)	98 (45.2)	
Grade of malignancy (G) G1	47 (6.4)	34 (72.5)	13 (27.5)	0.0607	28 (59.6)	19 (40.4)	**0.0183**
G2	548 (75.2)	456 (83.2)	92 (16.8)		270 (49.3)	278 (50.7)	
G3	134 (18.4)	117 (87.3)	12 (12.7)		83 (61.9)	51 (38.1)	

NSCLC—non-small cell lung cancer; AC—adenocarcinoma, SCC—squamous cell carcinoma; significance in bold.

**Table 2:** Associations of irisin expression level with clinicopathological characteristics in patients with NSCLC.

NSCLC	*P* Value (U-Mann–Whitney Test)			*Mean* Value *± SD*	
	Cancer Cells	Stromal Cells		Cancer Cells	Stromal Cells
Lymph Nodes			Lymph Nodes		
N0 vs. N1	0.0607	0.1477	N0	2.2 ± 0.13	4.8 ± 0.16
N0 vs. N2	**0.0124**	0.4401	N1	1.8 ± 0.20	5.1 ± 0.27
N1 vs. N2	**0.0011**	0.1502	N2	2.8 ± 0.28	4.6 ± 0.27
Tumour Size			Tumour Size		
T1 vs. T2	0.4377	**0.0228**	T1	2.3 ± 0.22	4.4 ± 0.24
T1 vs. T3–4	**0.0215**	0.0719	T2	2.3 ± 0.15	5.1 ± 0.19
T2 vs. T3–4	**0.0087**	0.3162	T3–4	1.9 ± 0.17	4.9 ± 0.23
Stage			Stage		
I vs. II	**0.0275**	**0.0023**	I	2.3 ± 0.17	4.4 ± 0.20
I vs. III–IV	0.5178	0.3322	II	2.0 ± 0.17	5.4 ± 0.21
II vs. III–IV	0.1637	**0.0324**	III–IV	2.3 ± 0.19	4.7 ± 0.21
Metastases			Metastases		
M0 vs. M1b	0.8822	**0.0054**	M0	2.2 ± 0.10	4.8 ± 0.12
			M1b	1.8 ± 0.82	9.0 ± 1.12

Abbreviations: NSCLC—non-small cell lung cancer; significance in bold.

**Table 3:** Univariate and multivariate Cox proportional hazards analysis in 729 patients with NSCLC.

*Clinicopathological Parameters*		*NSCLC*	
		Univariate Analysis HR (95% CI) p	Multivariate Analysis HR (95% CI) p
Age ≤60 vs. >60		1.25 (1.02–1.54) **0.0292**	1.35 (1.10–1.66) **0.0039**
Sex Male vs. Female		1.39 (1.11–1.75) **0.0045**	1.28 (1.02–1.61) **0.0343**
Smoking History Yes vs. No		1.31(0.97–1.76) 0.0702	
Pt T1–T2 vs. T3–T4		1.85 (1.52–2.25) **<0.0001**	1.49 (1.17–1.88) **<0.0001**
pN N0 vs. N+		1.78 (1.47–2.16) **<0.0001**	1.44 (1.12–1.85) **0.0029**
Grade G1 vs. G2–G3		1.19 (0.80–1.77) 0.3869	
Stage I–II vs. III–IV		2.09 (1.72–2.55) **<0.0001**	1.39 (1.04–1.85) **0.0233**
Irisin Cancer Cells <25% vs. ≥25%		1.05 (0.77–1.43) 0.7535	
Irisin Stromal Cells <25% vs. ≥25%		1.33 (1.08–1.63) **0.0054**	1.30 (1.06–1.60) **0.0111**
Ki-67 <25% vs. ≥25%		0.94 (0.77–1.14) 0.5464	
TTF-1 <25% vs. ≥25%		1.04 (0.86–1.27) 0.6235	
p63 <25% vs. ≥25%		0.85 (0.70–1.03) 0.0998	
TTF-1 <25% vs. ≥25%	0.98 (0.82–1.19) 0.1572		

Abbreviations: HR—hazard ratio, CI—confidence interval, NSCLC—non-small cell lung cancer; significance in bold.
